# A Smart Chair to Monitor Sitting Posture by Capacitive Textile Sensors

**DOI:** 10.3390/ma16134838

**Published:** 2023-07-05

**Authors:** Marc Martínez-Estrada, Tiina Vuohijoki, Anja Poberznik, Asif Shaikh, Johanna Virkki, Ignacio Gil, Raúl Fernández-García

**Affiliations:** 1 Departament of Electronic Engineering, Universitat Politecnica de Catalunya, ESEIAAT, Colom 1, 08222 Terrassa, Spain; ignasi.gil@upc.edu (I.G.); raul.fernandez-garcia@upc.edu (R.F.-G.); 2 Faculty of Information Technology and Communication Sciences, Tampere University, 33720 Tampere, Finland; tiina.vuohijoki@tuni.fi (T.V.); asif.shaikh@tuni.fi (A.S.); johanna.virkki@tuni.fi (J.V.); 3 Faculty of Technology, Satakunta University of Applied Sciences, 28130 Pori, Finland; anja.poberznik@samk.fi

**Keywords:** office chair, smart textile, embroidery, sensor, textile, presence

## Abstract

In this paper, a smart office chair with movable textile sensors to monitor sitting position during the workday is presented. The system consists of a presence textile capacitive sensor with different levels of activation with a signal conditioning device. The proposed system was integrated into an office chair to detect postures that could provoke musculoskeletal disorders or discomfort. The microcontroller measured the capacitance by means of a cycle count method and provided the position information in real time. The information could be analysed to set up warnings to prevent incorrect postures or the necessity to move. Five participants assumed a series of postures, and the results showed the workability of the proposed smart chair. The chair can be provided as a new tool for companies, hospitals, or other institutions to detect incorrect postures and monitor the postures of people with reduced mobility. This tool can optimise control procedures or prevent occupational risks.

## 1. Introduction

In sedentary occupations such as office work, prolonged static sitting poses a health risk for developing musculoskeletal disorders and cardiovascular, metabolic, and cognitive issues [1,2]. A good workstation consists of suitable furniture and (technical) equipment, optimal lighting, good air quality, and diminished noise disturbances [3]. Adjusting the workstation and chair setup to an ergonomically optimal position may reduce musculoskeletal risks for pain and discomfort of the lower back, elbows, fingers, and legs [4]. The literature often suggests an optimal office sitting posture where one should sit straight, yet following the natural spinal curvature, with feet flat on the floor, hips as far back in the chair as possible and knees in line with the hips. Elbows should be flexed at 90 degrees at the side of your body, shoulders relaxed, and wrists in a neutral position. The monitor should be directly in front at arm’s length, with the top of the screen at eye level to prevent neck strain [3,5].

However, there has been some controversy in the literature regarding the most ideal sitting posture or whether it even exists. The long-standing belief of sitting “as upright as possible” has been slowly replaced by the concept of “dynamic” or “active sitting” where sitting positions are changed as often as possible [6,7]. Yet again, a literature review [8] suggests that there is no ideal sitting posture, and for preventing low back pain, one should sit with a preferred lumbar lordosis and, in addition, move regularly. Frequent position changes for office workers, which include working in a standing position and moving regularly, are usually recommended by ergonomics experts and physiotherapists to avoid work-related discomfort and injuries [9].

People have adopted their own ways of sitting that are natural and comfortable for them, and the same sitting posture that feels good to someone might feel uncomfortable to another. In fact, eight main sitting postures have been identified in the literature [10,11]: upright sitting, slumped sitting, leaning forward, leaning backwards, leaning left, leaning right, right leg crossed, and left leg crossed. Often the users are not aware of the postures that they have adopted [12].

Ergonomic office chairs have been designed to help achieve optimal posture with their shape, adjustable height, armrests, adjustable reclining backrest and additional lumbar spine support [3]. Users, however, do not utilise all the adjustments and thus do not use the office chair in the optimal ergonomic way [12], either due to a lack of knowledge or simply their habitual sitting positions. A feedback system integrated into the chair may help tackle this issue and guide the user towards an optimal sitting posture behaviour.

The works of [13,14,15,16,17] analysed some feedback systems on office chairs. One of these works revised [14] presented an office chair with electronic distance sensors and pressure sensors for real-time posture classification. The distance sensors were installed on the chair’s back; meanwhile, the pressure sensors were installed on the seat. The chair was capable of obtaining information about people’s sitting positions. This information was taken and analysed to classify the positions automatically by a neuronal network (machine learning). The inconveniences of that office chair were, on one hand, that the distance sensors integrated into the backrest needed to be on the surface of the foam, decreasing the comfort. On the other hand, the pressure sensor could not provide information about raised body parts which did not apply pressure over the seat.

The ergonomic chair with Internet-of-things (IoT) capabilities presented in [13] was also designed with a common electronic force sensor. Despite the fact that they could obtain different levels of pressure over the force sensor, they were only distinguishing whether pressure was detected or not. A further analysis of the position was not possible because the system’s possibilities were reduced to a binary response. Related to [16], the feedback system had only two sensors on the backrest. They could not differentiate between high- or low-back displacements because both sensors covered the backrest surface.

A feedback system should be designed to be naturally integrated into the chair. Ergonomic chairs are commonly covered with textiles, which could be the ideal substrate to integrate sensors on. Electronic sensors are installed into the chair foam or underneath, which affects the user’s comfort and makes the replacement of the sensors difficult. Therefore, this solution would be unfeasible for the feedback system. Chair upholstery is easier to replace if the textile sensors fail.

Based on the reasons presented, capacitive textile sensors were chosen as the sensors for the feedback system. Due to the ability related to the permittivity change detection around it, a capacitive sensor provides an opportunity to obtain, with a single sensor, distance and presence data; meanwhile, for conventional electronic sensors, two different sensors are needed, as it shown in the literature.

To integrate the sensors into the chair fabric, there are plenty of textile processes used at the present time. Nevertheless, to produce a system that accomplishes the requirements, i.e., fast prototyping and low-cost production, an embroidery method was selected. Embroidery is commonly used in commercial garments and furniture and is thus well known to people, which is an asset when designing unobtrusive technology.

To produce a capacitive textile sensor, it is necessary to use conductive yarn, which is nowadays commercialised by some yarn companies, such as Shieldex [18] or Bekaert [19]. These yarns have conductive properties, which allow the integration of a sensor within, but also textile properties, which are derived from the manufacturing process to be unnoticeable by the user.

To obtain the data from the capacitive textile sensors, a microcontroller was connected to the sensors. The microcontroller analysed and organised the data of different textile sensors to store and then send them to a database or visualisation software. These real-time data provided an information source which reacted when it detected a bad position that could provoke disorders and notified the user to take preventive actions. This feedback system could be provided as a tool for companies to promote their workers’ health, or for hospitals to improve their wheelchairs’ usability and their attention to people with reduced mobility.

The novelty of this work is the design and production of a textile sensor which could be used as an alternative to the conventional electronic sensor used in the literature to track the body positions on an office chair. As the sensors are completely integrated into the upholstery of the chair, their impact on the user comfort and chair structure is lower than the conventional electronic sensors previously used in the literature. A case study with embroidered capacitive sensors that aims to track body positions from an office chair is presented next.

## 2. Materials and Methods

### 2.1. Office Chair Preparation

**Figure 1:**
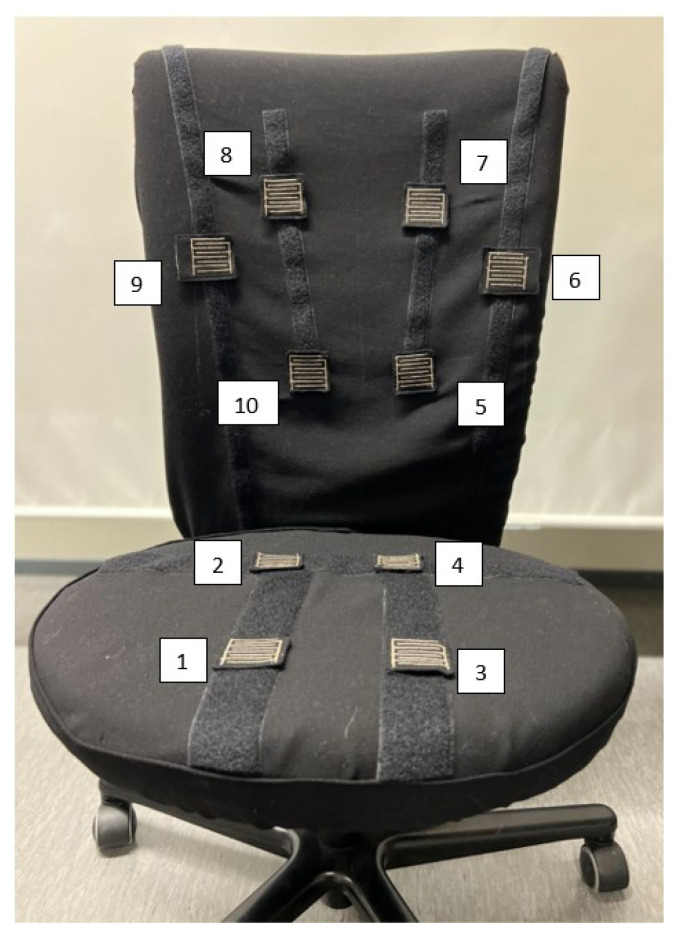
Smart chair with sensor distribution and number identification.

The locations of the sensors were identified to correspond to the optimal sitting ergonomics as identified in the previous section. The smart chair and the sensor distribution are shown in Figure 1. The sensors were positioned at the spots on the chair where the user’s body comes into contact with the chair.

The following sensor locations were identified: (i) bilaterally on the lower back (to promote a straight posture with the hips as far back in the chair as possible); (ii) bilaterally on the upper back just beneath the shoulder blades to promote the user’s contact with the backrest of the chair and to prevent slumped sitting and forward lean posture; (iii) bilaterally on the sides of the midback to prevent excessive leaning to the side; and (iv) bilaterally on the buttocks and on the back of the thighs to ensure contact with the seat and to discourage crossing the legs.

**Figure 2:**
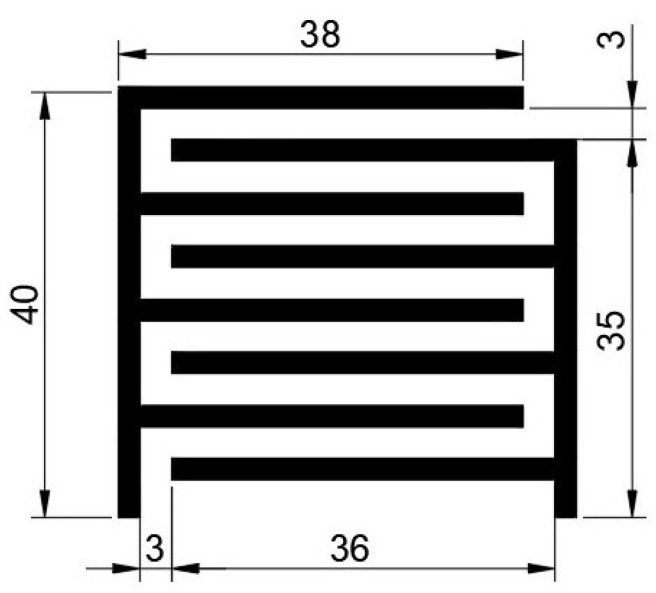
Interdigitated sensor geometry (dimensions in mm).

To detect the user’s sitting position on the chair, ten interdigitated capacitive embroidered sensors were produced. The interdigitated pattern can be observed in Figure 2.

The decision to use an interdigital structure was made because of two conditions: the capacitive sensor integrated into one layer and the response obtained from this well-known structure. The capacitance value was determined by the dimensions of the pattern and properties of the materials around them. Because the sensor was used to distinguish between certain position circumstances, dimension parameters acquired a higher importance than other parameters. Dimensions should be big enough to characterise different sitting positions and levels of body proximity but not so big that the capacitive-value-derived experiments cause an excessive increase or occupy too much space. Bigger sensors could produce misunderstandings in positional distinction. To obtain an approximation for the capacitance of each sensor, the dimensions were determined by the following equations [20].

(1) $$C_{RH}=\frac{\varepsilon_{RH}\cdot 10^{-3}}{18\pi }\frac{K(k)}{K^{\prime }\left(k\right)}l\left(N-1\right)\left(pF\right)$$

(2) $$\frac{K(k)}{K^{\prime }\left(k\right)}=\frac{\pi }{ln2\frac{1+\sqrt{k^{\prime }}}{1-\sqrt{k^{\prime }}}}$$

(3) $$k=tan^{2}\left(\frac{a\pi }{4b}\right)$$

(4) $$a=\frac{W}{2}$$

(5) $$k^{\prime }=\sqrt{1-k^{2}}$$

(6) $$b=\frac{W+S}{2}$$

where *l* is in microns (10${}^{-3}$ mm), *N* is the number of fingers, and $\varepsilon_{RH}$ is the effective dielectric constant of the material substrate, *W* is the finger width, and *S* is the space between fingers. The result obtained is the capacitance of the interdigital sensor in *pF*.

**Figure 3:**
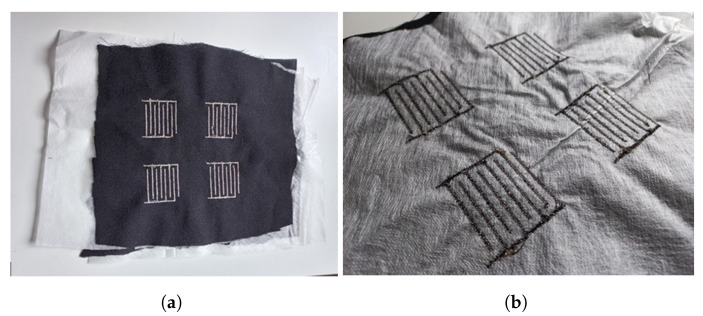
PreCaTex sensor embroidered with the Husqvarna Viking Designer Ruby Royale. (**a**) Top side of the sensor. (**b**) Bottom side of the sensor.

After obtaining this balance, the sensor was embroidered on a Husqvarna Viking Designer Ruby Royale sewing machine. The pattern was designed in AutoCAD and introduced as a JPEG file in embroidery machine software. The embroidery parameters, such as stitch density or embroidery pattern, were selected at that stage. A Shieldex 110/34 dtex 2-ply conductive yarn [18] was inserted as the top thread during the embroidering process. A common cotton yarn with a similar density was inserted from the bottom. The final substrate used was a 100% wool crepe fabric. To embroider the sensor on the substrate, a support nonwoven fabric was used to avoid movement during the manufacturing process. The support fabric could be removed afterwards by being diluted in water. The final presence textile capacitive sensor (PreCaTex), embroidered can be observed in Figure 3.

A commonly available office chair without armrests was used for this prototype. The chair was covered with 100% wool crepe fabric to create a surface to which the sensors were attached. Sensor attachment was designed to be movable. People are individually shaped and sized; therefore, the prototype needed to be adjustable to the user’s attributes.

There were several options for attaching the PreCaTex sensors to be movable, such as attaching them to the cover, using magnets to hold them in the right location, or utilising Velcro, which allows the easy relocation of the sensor. We decided to use the latter.

Most of the sensors were sewn individually, and to add possibilities to inspect the optimal locations, a couple of sensors were sewn in sets of twos. The embroidered fabric was face up on the office chair. The Velcro fabric (hook side) covered the bottom of the sensor flap, thus connecting to the Velcro (loop side) placed on the cover.

**Figure 4:**
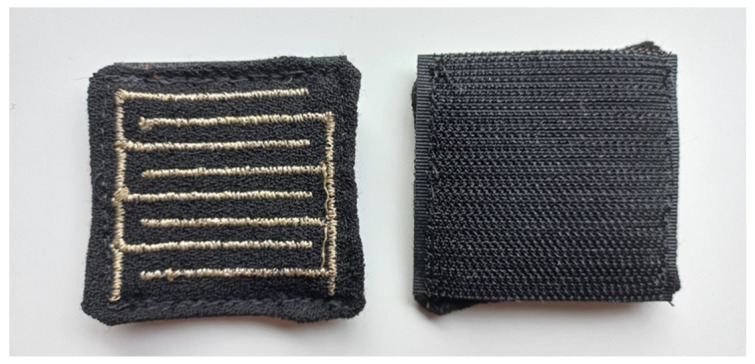
Ready-made sensor from both sides.

The embroidered sensor pieces were cut to suitable sizes, i.e., 5.5 cm × 5.0 cm, and each sensor piece’s sideline was folded to the reverse side, then pinned and neatened with hand-basted stitching. Afterwards, the Velcro was cut into the same-size pieces as the front ones, 5.5 cm × 5.0 cm, which matched the embroidered pieces, and then sewn together by topstitching. Figure 4 shows the ready-made sensor.

### 2.2. Test Procedure

**Figure 5:**
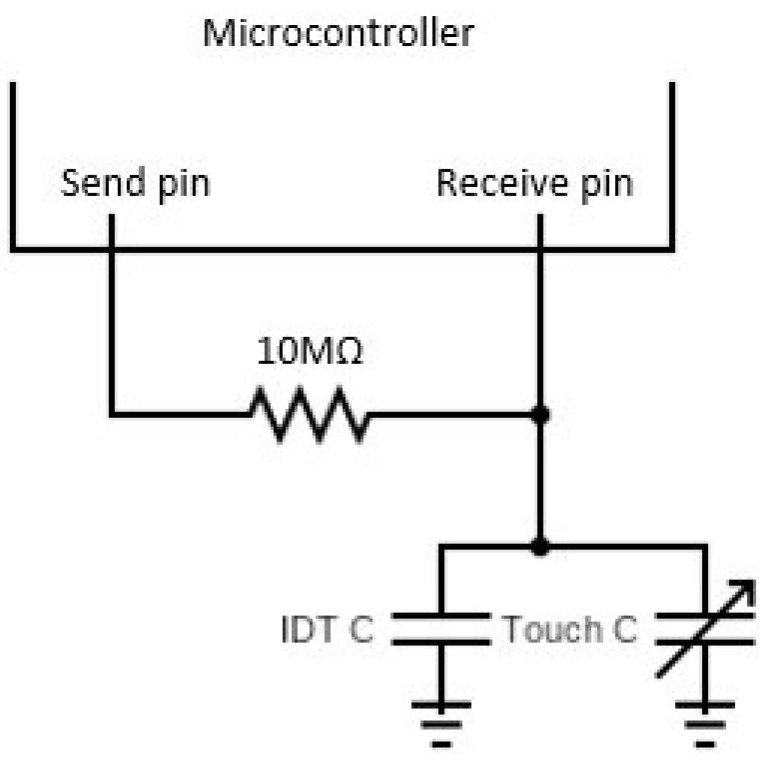
Measurement circuit of each individual sensor.

To gather the data from the PreCaTex sensors, an ATmega2560 microcontroller was used. Each individual sensor was connected to the microcontroller, which was in charge of measuring the capacitance of the sensor. To evaluate the data from the PreCaTex sensor, a cycle count method was used (Figure 5). Two microcontroller pins were required, one for the send pin and one for the receive pin. These pins were connected to a 10 MΩ resistance between them. On the receive pin’s side, one sensor electrode was connected to measure the tension. The measurement process started by setting up a tension of 5 V in the send pin. Then, the current went through the resistance and started charging the sensor capacitor. In each cycle, the microcontroller programming compared the tension measured in the received pin with the sensor in the send pin. If there was no match between them, the software program increased the cycle count by one and started a new cycle. Each cycle where the program evaluated the receive pin to compare it with the send pin was counted as a new cycle. When the sensor capacitor was fully charged, the receive pin was at 5 V, and the program stopped counting the cycles. The number of cycles counted was used as the value of the sensor. This measurement process was performed for each sensor constantly. A complete microcontroller scheme with every sensor circuit is available in Supplementary Files Figure S1. Also, the microcontroller programming for the correct measurement is presented in Supplementary Files Text S1.

The measurement methodology explained provided the opportunity to obtain information which could be used to distinguish between levels of pressure. These different levels of pressure were expected due to the body weight distribution. The more pressure applied with the body, the higher the capacitance value the sensor took. The capacitive sensor value variation was related to the distance between the sensor surface and the person’s skin, which provoked an increase in the permittivity in the sensor’s surroundings. That distance had a minimum value, which was found where the maximum pressure was applied on the sensor surface and the clothes in contact with it were compressed. In Table 1, different levels of activation are presented.

The first sensor level indicating contact between the sensor and the body is marked with a red dashed line in each figure in the Results section.

As this was an initial proof of concept, five of the authors were taken as volunteers to participate in the technical evaluation of the smart chair, in addition to the design works. For future studies, the number of participants should be increased in order to search and study thoroughly the data from PreCaTex sensors’ responses. All the measurements were taken in office settings, and the researcher adjusted the chair height to meet each participant’s individual fit, meaning that the participant’s feet were in contact with the floor and the participant’s buttock area was located as close to the back of the chair as possible. Researchers asked the participants to focus on exaggerated positions to obtain accurate data from the capacitive sensor. The capacitive sensors were confirmed to meet the desired locations corresponding to the participant’s physical markers. In other words, the upper sensors met the scapula (shoulder blade), and the seat’s sensors meet the ischium bones (the bottom bones that touch the seat) and both thighs. There were only slight changes made to the capacitive sensor locations between the measured participants.

**Figure 6:**
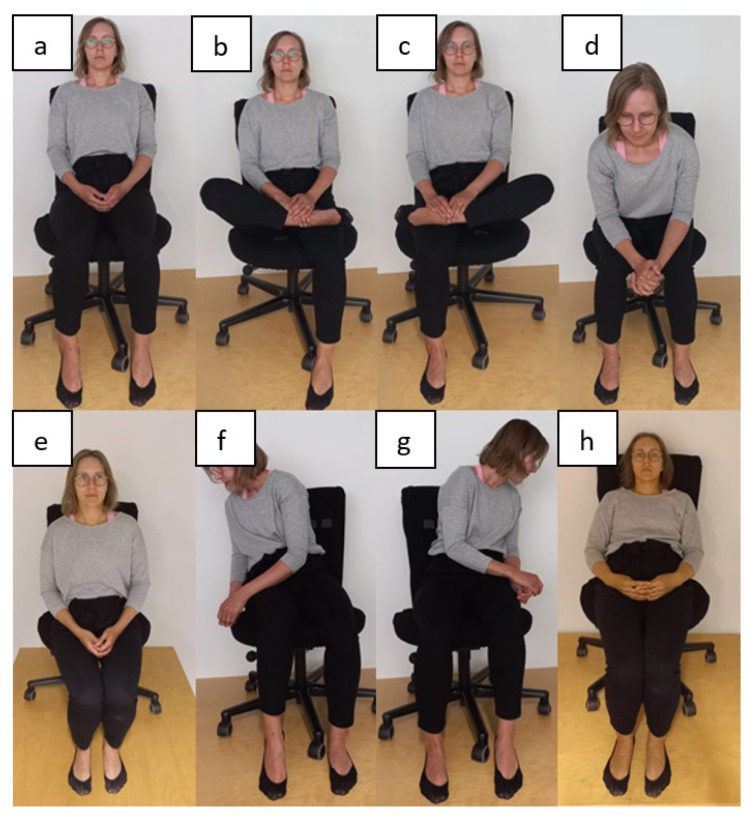
Measured sitting positions during the smart chair test. (**a**). Ergonomic posture, (**b**). Right leg crossed, (**c**). Left leg crossed, (**d**). Detach from the backrest, (**e**). Sit on the edge, (**f**). Lean to the right, (**g**), Lean to the left and (**h**). Lean back and sit on the edge.

The position measurement process focussed on the evaluation of eight sitting positions. The positions selected included the optimal sitting position for work, which was defined by ergonomic studies [3,5], and other common sitting positions found in related work [13,14]. Additional common sitting positions were included in the process. These positions are presented in Figure 6.

The preselected measurement positions were explained and demonstrated by the researcher and practised by participants before the official measurement. Each of the participants completed the cycle of positions in a compressed time between 2 to 3 min.

**Figure 7:**
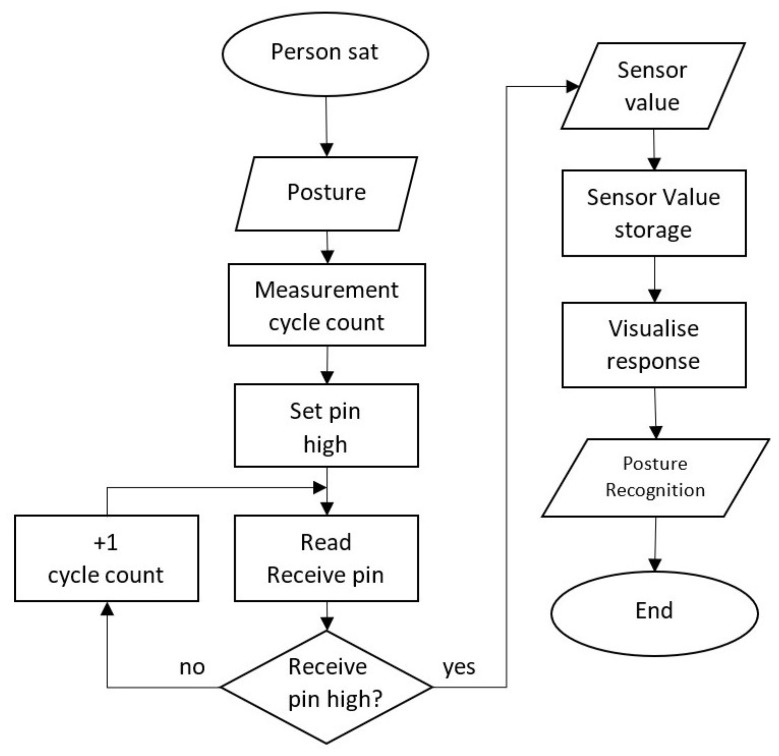
Flux diagram of the test procedure.

Figure 7 shows the flux diagram of the complete test procedure. To sum up, a participant sat on the chair, where the 10 sensors integrated were read by the ATmega2560 using the cycle count method. The microcontroller assigned each cycle count value to each sensor. The individual sensor values were sent and stored on the researcher’s computer to be evaluated and visualised in real time. The values were used to proceed with the posture recognition The aim of this proof of concept was to demonstrate that the capacitive sensor was capable of monitoring the sitting position and evaluating it.

## 3. Results and Discussion

For the evaluation of the proof of concept, five individual cycles of measurement with five different persons were compared. They were dressed in different kinds of jeans and shirts or T-shirts. Homogeneous clothing was expected to decrease the variability of the sensor values during the measurement and facilitate the analysis of the measurement.

The smart chair was prepared to obtain data from different positions for each person. PreCaTex sensors were placed and connected to the microcontroller. During the test, different levels of activation were expected (as mentioned in Section 2).

The smart chair data are presented for each position in a figure, which includes the sensor values for every participant for that particular position. Values for each PreCaTex sensor were compared between the participants. The values for general and individual characteristics of each position and participant were analysed. Complete data tables are available in Supplementary Files for every participant from Tables S1–S5.

### 3.1. The Ergonomic Posture

**Figure 8:**
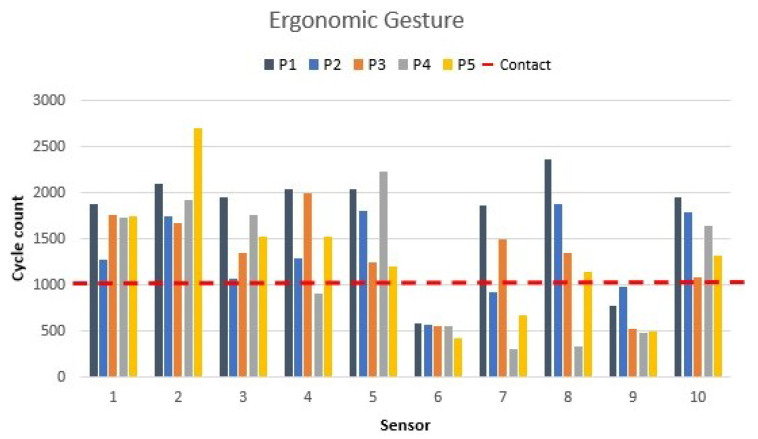
The ergonomic posture sensor values as shown in Figure 6a.

Firstly, participants were asked to assume the ergonomic posture. This is the position that should not create a disorder risk or tension all over the body. Data from the ergonomic position are shown in Figure 8.

The data from the ergonomic position for all five participants were compared. The buttock position was monitored by sensors 1 to 4. The four sensors were activated during the ergonomic position, acquiring values around 1000 cycles.

The position of the legs could increase or decrease the value. A sensor value lower than 1000 indicated that the legs could be displaced from the sensor.

The level of activation of the backrest sensors depended on how the participants had their upper and lower back positioned in the chair. The graph shows that side sensors 6 and 9 did not reach the red dashed line, which indicated that the sensor was detecting the body’s proximity but was not in contact with the sensor.

Participant 4 did not activate the upper-back sensors (7 and 8), which may indicate that their back curvature did not permit them to be close enough to the sensor. For the remaining participants, that sensor value was activated and reached the dashed line.

Three participants (2, 4, and 5) showed a noticeable asymmetrical distribution to the right. The differences observed for the sensor values were calculated as ΔS=(S2−S4). The result provided an estimation of how much more pressure was applied to the right side. Participant 2 had ΔS=456, participant 4 had ΔS=152, and participant 5 had ΔS=1170, which was the highest asymmetrical value observed. However, participant 3 showed an asymmetrical position to the left. The value ΔS=−325 was obtained. The negative value indicated that a higher value was obtained in sensor 4, which corresponded to the left side.

### 3.2. Right and Left Leg Crossed

**Figure 9:**
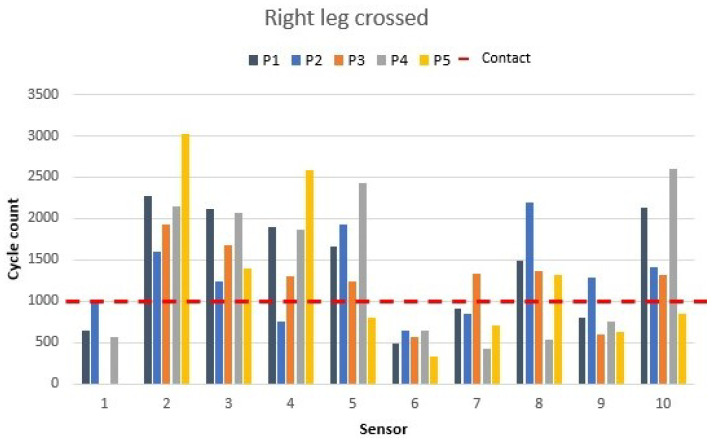
Crossed right leg’s sensor values as shown in Figure 6b.

Consecutively, right leg crossed and left leg crossed were analysed. These are common positions used by office workers to provide distension to their bodies. First, participants were asked to cross their right leg over the left leg. The right leg sensor values are shown in Figure 9.

During the measurement of that position, values close to 0 or lower than 500 were expected on the right-edge seat sensor (sensor 1), which was the sensor in contact with the right leg. Four participants, all but participant 2, matched this condition and showed how the pressure remained only in the left-edge seat sensor (sensor 3). Participant 2 increased the pressure over sensors 8 and 9, which corresponded to the right-side upper back and side middle back. The upper-back sensor (sensor 8) increased its value by 308 cycles due to the pressure, and the middle back sensor (sensor 9) showed an increase of 306 cycles. Participant 4 held all the pressure changes on the lower-back sensor (sensor 10). The increase of 946 cycles indicates that the pressure applied changed the level of detection of the sensor to close-contact high pressure. Participant 1’s right leg movement caused a decrease in the pressure on their high back, which was displayed on the high backrest sensors (sensors 8 and 9).

**Figure 10:**
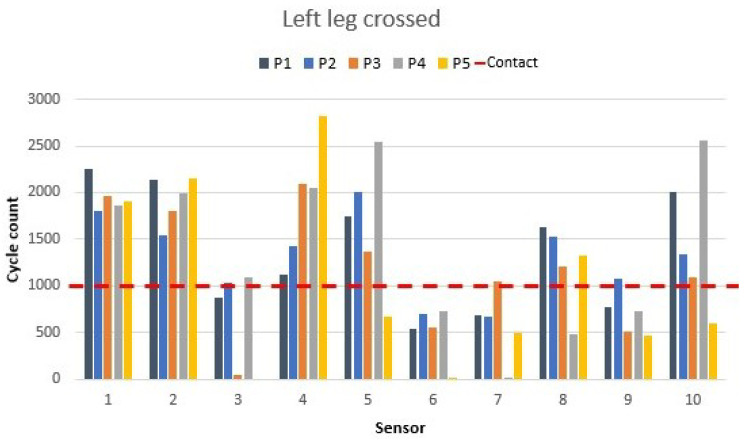
Crossed left leg’s sensor values as shown in Figure 6c.

To view when the leg crossed changed from left to right, the pressure redistribution can be observed in Figure 10.

The most noticeable change came from the leg change. The right-edge seat sensor (sensor 1) reached the value that it had during the ergonomic position. However, the left-edge seat sensor (sensor 3) values decreased due to the left leg movement. As in the previous situation, the values in sensor 3 decreased lower than the 1000 cycle count. Participants 1, 2, and 3 showed values close to the detection line; their flexibility or leg size did not let them detach the leg enough from the sensor. The backside’s back seat sensor values reached a higher value when the leg from the side was crossed, which was due to the increased contact or pressure during the movement. This fact was more observed in the values of participant 5 in both positions. During the right leg cross, the sensor 2 value increased to 331 cycles, but for the left leg situation, the cycle count increased to 1290.

The left backrest sensor values exchanged some pressure with the right backrest sensors. That pressure change was noticeable in sensor 5. Participant 4’s left low-middle backrest sensors (sensors 6 and 7) decreased significantly during movement, especially sensor 6 with a zero-count cycle.

The crossed leg position showed how the smart chair could monitor the movement and detect the pressure distribution depending on which leg was crossed.

### 3.3. Detachment from the Backrest

**Figure 11:**
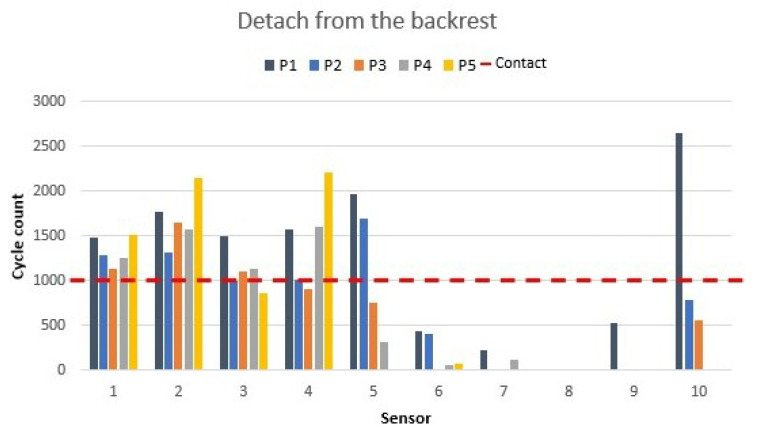
Detaching from the backrest sensor values as shown in Figure 6d.

The movement of detaching the back from the seat is performed by workers when they are doing tasks under pressure or focusing too much on the screen they are working on. Position values are shown in Figure 11.

The movement of detaching the back starting from the ergonomic position showed how the high backrest sensors (7 and 8) and side backrest sensors (6 and 9) were deactivated because of the back movement to the front. The values of the low backrest sensors 5 and 10 depended on the back movement and whether the back was detached when the movement was performed. Here, we can observe that participants 1, 2, and 3 did not move their lower backs as much as participants 4 and 5, who moved their backs away from the backrest in this position.

The seat sensor values were similar to the ergonomic position values, but some pressure change was observed.

### 3.4. Sitting on the Edge of the Chair

**Figure 12:**
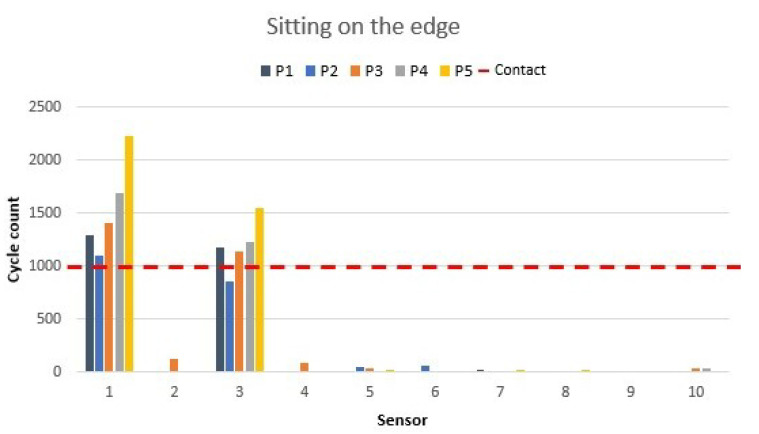
Sitting on the edge of the chair sensor values as shown in Figure 6e.

Sitting on the edge of the chair is one of the positions that some people take when sitting for a short time or just before standing up. The values of this position are presented in Figure 12. This position activated only the edge seat sensors 1 and 3. The rest of the sensors were not activated. This position should be defined in the microcontroller with a clock, which would activate an alarm if there was no change in 10 s.

### 3.5. Right and Left Leaning

**Figure 13:**
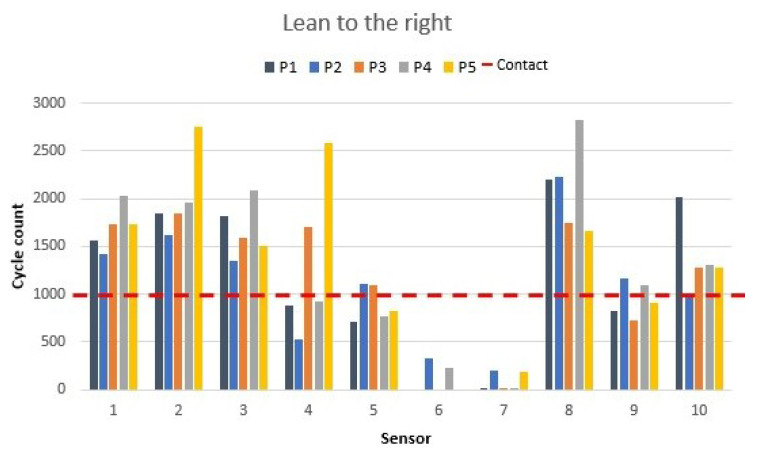
Right-leaning sensor values as shown in Figure 6f.

Right and left leaning can happen if a person is about to fall off a chair or if the sitting position is very asymmetrical. Thus, two leaning-to-the-side situations were measured. Firstly, the values of a person leaning to the right are presented in Figure 13.

When the participants leaned to the right side, sensors 6 and 7 were deactivated; their values reached close to a zero cycle count. The values of sensor 9 (middle backrest side sensor) increased due to the body leaning to the right. The increase observed was from 200 to 500 cycles for each participant. Sensor 8 displayed an increase in its value of 200–500 cycles, which was the consequence of all the pressure lying on the right side of the chair. For participant 4, sensor 8 displayed the biggest increase, which meant 2500 cycles more because the participant was unable to push their back close enough to the sensor when in the ergonomic position.

**Figure 14:**
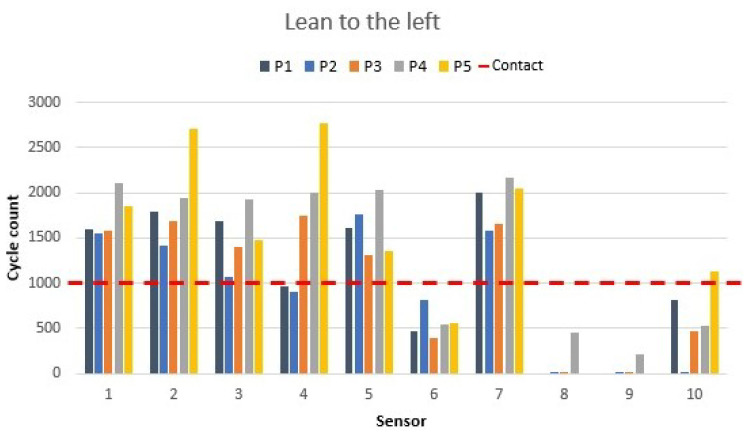
Left-leaning sensor values as shown in Figure 6g.

Following the procedure, the participants leaned to the left, and sensor responses were the opposite of the previous position. The sensor values for left leaning are shown in Figure 14.

Sensors 8 and 9 were deactivated due to the movement to the left. Sensors 6 and 7, which were off previously, became activated. Some of the data taken showed that sensor 10 experienced some decrease in value, and in the meantime, sensor 5 increased in value due to the leaning, which resulted in all the participants having values higher than 1000 (contact detection). Seat sensors did not experience value changes during the movement of leaning to the sides. However, for both leaning situations, detection was demonstrated. Leaning detection could be more useful in other smart-chair applications, such as a wheelchair, where people with mobility disorders need to be monitored to avoid harm. Another application could be the detection of hyperactivity in kids. In these potential applications, feedback is essential for a smart chair to be useful.

### 3.6. Sitting on the Edge and Leaning the Body Back

**Figure 15:**
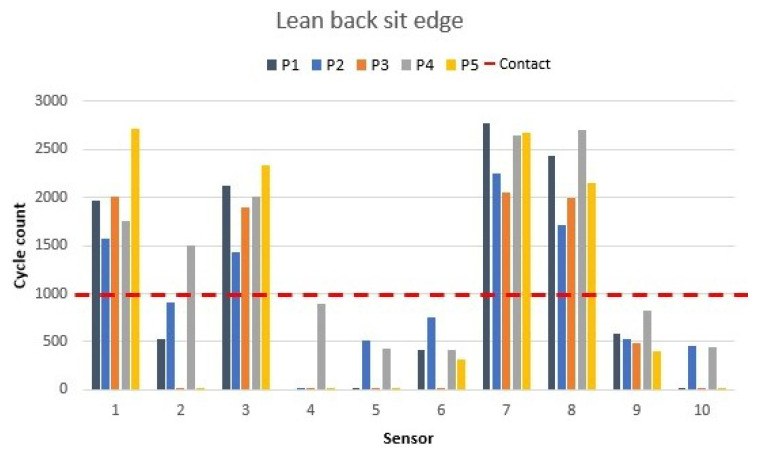
Sitting on the edge and leaning the body back sensor values as shown in Figure 6h.

This position is usually adopted by tired workers in an office situation. In this position, the lower back is detached from the backrest and moved forward while the upper back stays in contact with the chair. The back is thus not fully supported by the chair’s backrest and remains curved. These characteristics are observed in Figure 15.

Sensors 1 and 3 from the seat and 7 and 8 from the backrest were the ones activated in that position. Sensor 2 detected some body weight in some cases due to the position taken by the person during the measurement. A clear pattern was observed when that position was taken.

The values of sensors 1, 3, 7, and 8 were at their highest points, which indicated more pressure on them than in previous positions. Furthermore, the value difference between the sensors activated and the rest of the sensors was noticeable. The rest of the sensors showed values from 0 to 500 cycles, which corresponded to a sensor deactivation or body detection close-by but not in contact.

From the results obtained, it was demonstrated that the chair permitted the differentiation between different sitting positions and different levels of contact indicating proximity, contact, close contact, or high contact pressure. The chair provided a tool to correct the position, detect an asymmetrical gesture, and monitor movement behaviour. The information obtained by the smart chair may be useful in the prevention of low back pain or musculoskeletal disorders while sitting for a long time. The programming of the microcontroller at this stage focused on the ability to notify the user to change the sitting position or to stand up and move, in order to prevent musculoskeletal disorders.

Future work would be the complete integration of the sensor into the upholstery of the chair and different kinds of integration depending on the applications, such as wheelchairs or school chairs. Moreover, the alarm system needs to be studied according to ergonomics studies. The maximum sitting time, detecting the incorrect sitting position and correcting it, and notifying the user to stand up and move should be improved in the future. The measurement tests should be accompanied by video or images to obtain more precise information for the analysis of the sitting positions.

## 4. Conclusions

In this paper, we presented a detailed description of the initial prototype of an office chair with presence capacitive textile sensors (PreCaTex) detecting the user’s movement and positioning. The PreCaTex sensor could identify four levels of proximity and contact of the body; two of them were close-contact detection. Real-time data provided perfect information to the microcontroller to prepare warnings for the user, such as the need for movement or a poor position taken. It is essential to note the sensors’ low-cost and relatively effortless fabrication with a commercial embroidery machine. However, as this study was a proof of concept and tests were made in laboratory conditions, we still have many unanswered questions about the chair’s viability in real-life use. Nevertheless, after a more thorough investigation of the sensors’ features, we might be able to adopt this fabrication method in applications serving, for example, people with disabilities or impairments. If a similar kind of covering were placed on a wheelchair, it could help identify those body parts that are easily exposed to redundant pressure and insidiously cause skin traumas and discomfort.

## Figures and Tables

**Table 1:** Levels of activation of the sensor depending on the cycle count.

	Surrounding Detection	Contact	Close Contact (Pressure)	Close Contact (High Pressure)
Cycle count	±500	±1000	±1500	±2000

## Data Availability

No data available.

## Supplementary Materials

The following supporting information can be downloaded at: https://www.mdpi.com/article/10.3390/ma16134838/s1, Figure S1: Arduino mega circuit with sensors; Text S1: Microcontroller Programming; Table S1: P1 gesture measurement; Table S2: P2 gesture measurement; Table S3: P3 gesture measurement; Table S4: P4 gesture measurement and Table S5: P5 gesture measurement.
