# Supplementary material for: A Smart Chair to Monitor Sitting Posture by Capacitive Textile Sensors
Source: Materials (Basel). 2023 Jul 5;16(13):4838. doi: 10.3390/ma16134838 (PMC10343373; doi:10.3390/ma16134838)
Supplement: Supplementary file 1 [file materials-16-04838-s001.zip › Microcontroller programming.pdf]

Program 1

```
#include <CapacitiveSensor.h>

#include "Keyboard.h"

/*

 * CapitiveSense Library Demo Sketchr

 * Resistor effects sensitivity, experiment with values, 50K - 50M. Larger resistor values yield larger
sensor values.

 * Receive pin is the sensor pin - try different amounts of foil/metal on this pin

 */



CapacitiveSensor   s1 = CapacitiveSensor(4,2);        // 10M resistor between pins 4 & 2, pin 2 is sensor
pin, add a wire and or foil if desired

CapacitiveSensor   s2 = CapacitiveSensor(4,3);

CapacitiveSensor   s3 = CapacitiveSensor(4,5);

CapacitiveSensor   s4 = CapacitiveSensor(6,7);

CapacitiveSensor   s5 = CapacitiveSensor(6,8);

CapacitiveSensor   s6 = CapacitiveSensor(6,9);

CapacitiveSensor   s7 = CapacitiveSensor(10,11);

CapacitiveSensor   s8 = CapacitiveSensor(10,12);

CapacitiveSensor   s9 = CapacitiveSensor(10,13);

CapacitiveSensor   s10 = CapacitiveSensor(14,15);



long val1,val2, val3,val4,val5,val6,val7,val8,val9,val10;


void setup()

{

  Serial.println("Sensor 1, Sensor 2, Sensor 3, Sensor 4,Sensor 5, Sensor 6,Sensor 7, Sensor 8,Sensor 9,
Sensor 10 ,");

 //s1.set_CS_AutocaL_Millis(0xFFFFFFFF);
```

```
 //s2.set_CS_AutocaL_Millis(0xFFFFFFFF);
 //s3.set_CS_AutocaL_Millis(0xFFFFFFFF);
 // s4.set_CS_AutocaL_Millis(0xFFFFFFFF);
 // s5.set_CS_AutocaL_Millis(0xFFFFFFFF);
 // s6.set_CS_AutocaL_Millis(0xFFFFFFFF);
 // s7.set_CS_AutocaL_Millis(0xFFFFFFFF);
 // s8.set_CS_AutocaL_Millis(0xFFFFFFFF);
 // s9.set_CS_AutocaL_Millis(0xFFFFFFFF);
 // s10.set_CS_AutocaL_Millis(0xFFFFFFFF);
Serial.begin(9600);

}

void loop()
{

   val1 =  s1.capacitiveSensor(30);
   delay(10);
   val2 =  s2.capacitiveSensor(30);
   delay(10);
   val3 =  s3.capacitiveSensor(30);
   delay(10);
   val4 =  s4.capacitiveSensor(30);
   delay(10);
   val5 =  s5.capacitiveSensor(30);
   delay(10);
   val6 =  s6.capacitiveSensor(30);
   delay(10);
   val7 =  s7.capacitiveSensor(30);
   delay(10);
```

```
   val8 =  s8.capacitiveSensor(30);
   delay(10);
   val9 =  s9.capacitiveSensor(30);
   delay(10);
   val10 =  s10.capacitiveSensor(30);
   delay(10);


 Serial.print(",");
 Serial.print(val1);
 Serial.print(",");
 Serial.print(val2);
 Serial.print(",");
 Serial.print(val3);
 Serial.print(",");
 Serial.print(val4);
 Serial.print(",");
 Serial.print(val5);
 Serial.print(",");
 Serial.print(val6);
 Serial.print(",");
 Serial.print(val7);
 Serial.print(",");
 Serial.print(val8);
 Serial.print(",");
 Serial.print(val9);
 Serial.print(",");
 Serial.print(val10);
   delay(1000);                    // arbitrary delay to limit data to serial port
}
```

Library CapacitiveSensor

/*

CapacitiveSense.h - Capacitive Sensing Library for 'duino / Wiring

https://github.com/PaulStoffregen/CapacitiveSensor

http://www.pjrc.com/teensy/td_libs_CapacitiveSensor.html

http://playground.arduino.cc/Main/CapacitiveSensor

Copyright (c) 2009 Paul Bagder

Updates for other hardare by Paul Stoffregen, 2010-2016

vim: set ts=4:

Permission is hereby granted, free of charge, to any person obtaining a copy of this software and associated documentation files (the "Software"), to deal in the Software without restriction, including without limitation the rights to use, copy, modify, merge, publish, distribute, sublicense, and/or sell copies of the Software, and to permit persons to whom the Software is furnished to do so, subject to the following conditions:

The above copyright notice and this permission notice shall be included in all copies or substantial portions of the Software.

THE SOFTWARE IS PROVIDED "AS IS", WITHOUT WARRANTY OF ANY KIND, EXPRESS OR IMPLIED, INCLUDING BUT NOT LIMITED TO THE WARRANTIES OF MERCHANTABILITY, FITNESS FOR A PARTICULAR PURPOSE AND NONINFRINGEMENT. IN NO EVENT SHALL THE AUTHORS OR COPYRIGHT HOLDERS BE LIABLE FOR ANY CLAIM, DAMAGES OR OTHER LIABILITY, WHETHER IN AN ACTION OF CONTRACT, TORT OR OTHERWISE, ARISING FROM, OUT OF OR IN CONNECTION WITH THE SOFTWARE OR THE USE OR OTHER DEALINGS IN THE SOFTWARE.

*/

```
// ensure this library description is only included once
#ifndef CapacitiveSensor_h
#define CapacitiveSensor_h

#if ARDUINO >= 100
#include "Arduino.h"
#else
#include "WProgram.h"
#endif

// Direct I/O through registers and bitmask (from OneWire library)

#if defined(__AVR__)
#define PIN_TO_BASEREG(pin)             (portInputRegister(digitalPinToPort(pin)))
#define PIN_TO_BITMASK(pin)             (digitalPinToBitMask(pin))
#define IO_REG_TYPE uint8_t
#define DIRECT_READ(base, mask)         (((*(base)) & (mask)) ? 1 : 0)
#define DIRECT_MODE_INPUT(base, mask)   ((*((base)+1)) &= ~(mask), (*((base)+2)) &= ~(mask))
#define DIRECT_MODE_OUTPUT(base, mask)  ((*((base)+1)) |= (mask))
#define DIRECT_WRITE_LOW(base, mask)    ((*((base)+2)) &= ~(mask))
#define DIRECT_WRITE_HIGH(base, mask)   ((*((base)+2)) |= (mask))

#elif defined(__MK20DX128__) || defined(__MK20DX256__) || defined(__MK66FX1M0__) ||
defined(__MK64FX512__)
#define PIN_TO_BASEREG(pin)             (portOutputRegister(pin))
#define PIN_TO_BITMASK(pin)             (1)
#define IO_REG_TYPE uint8_t
#define IO_REG_ASM
#define DIRECT_READ(base, mask)         (*((base)+512))
#define DIRECT_MODE_INPUT(base, mask)   (*((base)+640) = 0)
```

```
#define DIRECT_MODE_OUTPUT(base, mask)  (*((base)+640) = 1)
#define DIRECT_WRITE_LOW(base, mask)    (*((base)+256) = 1)
#define DIRECT_WRITE_HIGH(base, mask)   (*((base)+128) = 1)

#elif defined(__MKL26Z64__)
#define PIN_TO_BASEREG(pin)           (portOutputRegister(pin))
#define PIN_TO_BITMASK(pin)           (digitalPinToBitMask(pin))
#define IO_REG_TYPE uint8_t
#define IO_REG_ASM
#define DIRECT_READ(base, mask)        ((*((base)+16) & (mask)) ? 1 : 0)
#define DIRECT_MODE_INPUT(base, mask)   (*((base)+20) &= ~(mask))
#define DIRECT_MODE_OUTPUT(base, mask)  (*((base)+20) |= (mask))
#define DIRECT_WRITE_LOW(base, mask)    (*((base)+8) = (mask))
#define DIRECT_WRITE_HIGH(base, mask)   (*((base)+4) = (mask))

#elif defined(__IMXRT1052__) || defined(__IMXRT1062__)
#define PIN_TO_BASEREG(pin)           (portOutputRegister(pin))
#define PIN_TO_BITMASK(pin)           (digitalPinToBitMask(pin))
#define IO_REG_TYPE uint32_t
#define IO_REG_ASM
#define DIRECT_READ(base, mask)        ((*((base)+2) & (mask)) ? 1 : 0)
#define DIRECT_MODE_INPUT(base, mask)   (*((base)+1) &= ~(mask))
#define DIRECT_MODE_OUTPUT(base, mask)  (*((base)+1) |= (mask))
#define DIRECT_WRITE_LOW(base, mask)    (*((base)+34) = (mask))
#define DIRECT_WRITE_HIGH(base, mask)   (*((base)+33) = (mask))

#elif defined(__SAM3X8E__)
#define PIN_TO_BASEREG(pin)           (&(digitalPinToPort(pin)->PIO_PER))
#define PIN_TO_BITMASK(pin)           (digitalPinToBitMask(pin))
#define IO_REG_TYPE uint32_t
```

```
#define IO_REG_ASM
#define DIRECT_READ(base, mask)        (((*((base)+15)) & (mask)) ? 1 : 0)
#define DIRECT_MODE_INPUT(base, mask)   ((*((base)+5)) = (mask))
#define DIRECT_MODE_OUTPUT(base, mask)  ((*((base)+4)) = (mask))
#define DIRECT_WRITE_LOW(base, mask)    ((*((base)+13)) = (mask))
#define DIRECT_WRITE_HIGH(base, mask)   ((*((base)+12)) = (mask))

#elif defined(__PIC32MX__)
#define PIN_TO_BASEREG(pin)           (portModeRegister(digitalPinToPort(pin)))
#define PIN_TO_BITMASK(pin)           (digitalPinToBitMask(pin))
#define IO_REG_TYPE uint32_t
#define IO_REG_ASM
#define DIRECT_READ(base, mask)        (((*(base+4)) & (mask)) ? 1 : 0)  //PORTX + 0x10
#define DIRECT_MODE_INPUT(base, mask)   ((*(base+2)) = (mask))           //TRISXSET + 0x08
#define DIRECT_MODE_OUTPUT(base, mask)  ((*(base+1)) = (mask))           //TRISXCLR + 0x04
#define DIRECT_WRITE_LOW(base, mask)    ((*(base+8+1)) = (mask))         //LATXCLR  + 0x24
#define DIRECT_WRITE_HIGH(base, mask)   ((*(base+8+2)) = (mask))         //LATXSET + 0x28

#elif defined(ARDUINO_ARCH_ESP8266)
#define PIN_TO_BASEREG(pin) ((volatile uint32_t*) GPO)
#define PIN_TO_BITMASK(pin) (1 << pin)
#define IO_REG_TYPE uint32_t
#define IO_REG_ASM
#define DIRECT_READ(base, mask) ((GPI & (mask)) ? 1 : 0) //GPIO_IN_ADDRESS
#define DIRECT_MODE_INPUT(base, mask) (GPE &= ~(mask)) //GPIO_ENABLE_W1TC_ADDRESS
#define DIRECT_MODE_OUTPUT(base, mask) (GPE |= (mask)) //GPIO_ENABLE_W1TS_ADDRESS
#define DIRECT_WRITE_LOW(base, mask) (GPOC = (mask)) //GPIO_OUT_W1TC_ADDRESS
#define DIRECT_WRITE_HIGH(base, mask) (GPOS = (mask)) //GPIO_OUT_W1TS_ADDRESS

#elif defined(ARDUINO_ARCH_ESP32)
```

```
#include <driver/rtc_io.h>
#define PIN_TO_BASEREG(pin)             (0)
#define PIN_TO_BITMASK(pin)             (pin)
#define IO_REG_TYPE uint32_t
#define IO_REG_BASE_ATTR
#define IO_REG_MASK_ATTR

static inline __attribute__((always_inline))
IO_REG_TYPE directRead(IO_REG_TYPE pin)
{
   if ( pin < 32 )
      return (GPIO.in >> pin) & 0x1;
   else if ( pin < 40 )
      return (GPIO.in1.val >> (pin - 32)) & 0x1;

   return 0;
}

static inline __attribute__((always_inline))
void directWriteLow(IO_REG_TYPE pin)
{
   if ( pin < 32 )
      GPIO.out_w1tc = ((uint32_t)1 << pin);
   else if ( pin < 34 )
      GPIO.out1_w1tc.val = ((uint32_t)1 << (pin - 32));
}

static inline __attribute__((always_inline))
void directWriteHigh(IO_REG_TYPE pin)
{
```

```
    if ( pin < 32 )
        GPIO.out_w1ts = ((uint32_t)1 << pin);
    else if ( pin < 34 )
        GPIO.out1_w1ts.val = ((uint32_t)1 << (pin - 32));
}

static inline __attribute__((always_inline))
void directModeInput(IO_REG_TYPE pin)
{
    if ( digitalPinIsValid(pin) )
    {
        uint32_t rtc_reg(rtc_gpio_desc[pin].reg);

        if ( rtc_reg ) // RTC pins PULL settings
        {
            ESP_REG(rtc_reg) = ESP_REG(rtc_reg) & ~(rtc_gpio_desc[pin].mux);
            ESP_REG(rtc_reg) = ESP_REG(rtc_reg) & ~(rtc_gpio_desc[pin].pullup |
rtc_gpio_desc[pin].pulldown);
        }

        if ( pin < 32 )
            GPIO.enable_w1tc = ((uint32_t)1 << pin);
        else
            GPIO.enable1_w1tc.val = ((uint32_t)1 << (pin - 32));

        uint32_t pinFunction((uint32_t)2 << FUN_DRV_S); // what are the drivers?
        pinFunction |= FUN_IE; // input enable but required for output as well?
        pinFunction |= ((uint32_t)2 << MCU_SEL_S);

        ESP_REG(DR_REG_IO_MUX_BASE + esp32_gpioMux[pin].reg) = pinFunction;
```

```
        GPIO.pin[pin].val = 0;
    }
}

static inline __attribute__((always_inline))
void directModeOutput(IO_REG_TYPE pin)
{
    if ( digitalPinIsValid(pin) && pin <= 33 ) // pins above 33 can be only inputs
    {
        uint32_t rtc_reg(rtc_gpio_desc[pin].reg);

        if ( rtc_reg ) // RTC pins PULL settings
        {
            ESP_REG(rtc_reg) = ESP_REG(rtc_reg) & ~(rtc_gpio_desc[pin].mux);
            ESP_REG(rtc_reg) = ESP_REG(rtc_reg) & ~(rtc_gpio_desc[pin].pullup |
rtc_gpio_desc[pin].pulldown);
        }

        if ( pin < 32 )
            GPIO.enable_w1ts = ((uint32_t)1 << pin);
        else // already validated to pins <= 33
            GPIO.enable1_w1ts.val = ((uint32_t)1 << (pin - 32));

        uint32_t pinFunction((uint32_t)2 << FUN_DRV_S); // what are the drivers?
        pinFunction |= FUN_IE; // input enable but required for output as well?
        pinFunction |= ((uint32_t)2 << MCU_SEL_S);

        ESP_REG(DR_REG_IO_MUX_BASE + esp32_gpioMux[pin].reg) = pinFunction;
```

```
    GPIO.pin[pin].val = 0;
  }
}

#define DIRECT_READ(base, pin)          directRead(pin)
#define DIRECT_WRITE_LOW(base, pin)     directWriteLow(pin)
#define DIRECT_WRITE_HIGH(base, pin)    directWriteHigh(pin)
#define DIRECT_MODE_INPUT(base, pin)    directModeInput(pin)
#define DIRECT_MODE_OUTPUT(base, pin)   directModeOutput(pin)
// https://github.com/PaulStoffregen/OneWire/pull/47
// https://github.com/stickbreaker/OneWire/commit/6eb7fc1c11a15b6ac8c60e5671cf36eb6829f82c
#ifdef  interrupts
#undef  interrupts
#endif
#ifdef  noInterrupts
#undef  noInterrupts
#endif
#define noInterrupts() {portMUX_TYPE mux =
portMUX_INITIALIZER_UNLOCKED;portENTER_CRITICAL(&mux)
#define interrupts() portEXIT_CRITICAL(&mux);}
//#warning, code is copied from "ESP32 OneWire testing"

#elif defined(__SAMD21G18A__)
// runs extremely slow/unreliable on Arduino Zero - help wanted....
#define PIN_TO_BASEREG(pin)             portModeRegister(digitalPinToPort(pin))
#define PIN_TO_BITMASK(pin)             (digitalPinToBitMask(pin))
#define IO_REG_TYPE uint32_t
#define IO_REG_ASM
#define DIRECT_READ(base, mask)         (((*((base)+8)) & (mask)) ? 1 : 0)
#define DIRECT_MODE_INPUT(base, mask)   ((*((base)+1)) = (mask))
```

```
#define DIRECT_MODE_OUTPUT(base, mask)  ((*((base)+2)) = (mask))
#define DIRECT_WRITE_LOW(base, mask)    ((*((base)+5)) = (mask))
#define DIRECT_WRITE_HIGH(base, mask)   ((*((base)+6)) = (mask))

#elif defined(__SAMD51__)
#define PIN_TO_BASEREG(pin)             portModeRegister(digitalPinToPort(pin))
#define PIN_TO_BITMASK(pin)             (digitalPinToBitMask(pin))
#define IO_REG_TYPE uint32_t
#define IO_REG_ASM
#define DIRECT_READ(base, mask)         (((*((base)+8)) & (mask)) ? 1 : 0) // IN
#define DIRECT_MODE_INPUT(base, mask)   ((*((base)+1)) = (mask)) // DIRCLR
#define DIRECT_MODE_OUTPUT(base, mask)  ((*((base)+2)) = (mask)) // DIRSET
#define DIRECT_WRITE_LOW(base, mask)    ((*((base)+5)) = (mask)) // OUTCLR
#define DIRECT_WRITE_HIGH(base, mask)   ((*((base)+6)) = (mask)) /// OUTSET

#elif defined(ARDUINO_NRF52_ADAFRUIT) || defined(ARDUINO_ARCH_NRF52840)

/*
Required for the Arduino Nano 33 BLE Sense to satisfy the compiler
as build.f_cpu is not defined in the boards.txt file.
The concept of F_CPU doesn't fully apply as mbed RTOS is used which uses preemption.
*/
#if defined(ARDUINO_ARCH_NRF52840) && !defined(F_CPU)
#define F_CPU 64000000L
#endif

#define PIN_TO_BASEREG(pin)             (0)
#define PIN_TO_BITMASK(pin)             digitalPinToPinName(pin)
#define IO_REG_TYPE uint32_t
#define IO_REG_ASM
```

```
#define DIRECT_READ(base, pin)          nrf_gpio_pin_read(pin)
#define DIRECT_WRITE_LOW(base, pin)     nrf_gpio_pin_clear(pin)
#define DIRECT_WRITE_HIGH(base, pin)    nrf_gpio_pin_set(pin)
#define DIRECT_MODE_INPUT(base, pin)    nrf_gpio_cfg_input(pin, NRF_GPIO_PIN_NOPULL)
#define DIRECT_MODE_OUTPUT(base, pin)   nrf_gpio_cfg_output(pin)

#elif defined(RBL_NRF51822)
#define PIN_TO_BASEREG(pin)             (0)
#define PIN_TO_BITMASK(pin)             (pin)
#define IO_REG_TYPE uint32_t
#define IO_REG_ASM
#define DIRECT_READ(base, pin)          nrf_gpio_pin_read(pin)
#define DIRECT_WRITE_LOW(base, pin)     nrf_gpio_pin_clear(pin)
#define DIRECT_WRITE_HIGH(base, pin)    nrf_gpio_pin_set(pin)
#define DIRECT_MODE_INPUT(base, pin)    nrf_gpio_cfg_input(pin, NRF_GPIO_PIN_NOPULL)
#define DIRECT_MODE_OUTPUT(base, pin)   nrf_gpio_cfg_output(pin)

#elif defined(__arc__)

#include "scss_registers.h"
#include "portable.h"
#include "avr/pgmspace.h"

#define GPIO_ID(pin)                    (g_APinDescription[pin].ulGPIOId)
#define GPIO_TYPE(pin)                  (g_APinDescription[pin].ulGPIOType)
#define GPIO_BASE(pin)                          (g_APinDescription[pin].ulGPIOBase)
#define DIR_OFFSET_SS                   0x01
#define DIR_OFFSET_SOC                          0x04
#define EXT_PORT_OFFSET_SS              0x0A
#define EXT_PORT_OFFSET_SOC                     0x50
```

```
/* GPIO registers base address */
#define PIN_TO_BASEREG(pin)             ((volatile uint32_t *)g_APinDescription[pin].ulGPIOBase)
#define PIN_TO_BITMASK(pin)             pin
#define IO_REG_TYPE                     uint32_t
#define IO_REG_ASM

static inline __attribute__((always_inline))
IO_REG_TYPE directRead(volatile IO_REG_TYPE *base, IO_REG_TYPE pin)
{
   IO_REG_TYPE ret;
   if (SS_GPIO == GPIO_TYPE(pin)) {
      ret = READ_ARC_REG(((IO_REG_TYPE)base + EXT_PORT_OFFSET_SS));
   } else {
      ret = MMIO_REG_VAL_FROM_BASE((IO_REG_TYPE)base, EXT_PORT_OFFSET_SOC);
   }
   return ((ret >> GPIO_ID(pin)) & 0x01);
}

static inline __attribute__((always_inline))
void directModeInput(volatile IO_REG_TYPE *base, IO_REG_TYPE pin)
{
   if (SS_GPIO == GPIO_TYPE(pin)) {
      WRITE_ARC_REG(READ_ARC_REG((((IO_REG_TYPE)base) + DIR_OFFSET_SS)) & ~(0x01 <<
GPIO_ID(pin)),
                    ((IO_REG_TYPE)(base) + DIR_OFFSET_SS));
   } else {
      MMIO_REG_VAL_FROM_BASE((IO_REG_TYPE)base, DIR_OFFSET_SOC) &= ~(0x01 << GPIO_ID(pin));
   }
}
```

```
static inline __attribute__((always_inline))
void directModeOutput(volatile IO_REG_TYPE *base, IO_REG_TYPE pin)
{
   if (SS_GPIO == GPIO_TYPE(pin)) {
      WRITE_ARC_REG(READ_ARC_REG(((IO_REG_TYPE)(base) + DIR_OFFSET_SS)) | (0x01 <<
GPIO_ID(pin)),
                        ((IO_REG_TYPE)(base) + DIR_OFFSET_SS));
   } else {
      MMIO_REG_VAL_FROM_BASE((IO_REG_TYPE)base, DIR_OFFSET_SOC) |= (0x01 << GPIO_ID(pin));
   }
}

static inline __attribute__((always_inline))
void directWriteLow(volatile IO_REG_TYPE *base, IO_REG_TYPE pin)
{
   if (SS_GPIO == GPIO_TYPE(pin)) {
      WRITE_ARC_REG(READ_ARC_REG(base) & ~(0x01 << GPIO_ID(pin)), base);
   } else {
      MMIO_REG_VAL(base) &= ~(0x01 << GPIO_ID(pin));
   }
}

static inline __attribute__((always_inline))
void directWriteHigh(volatile IO_REG_TYPE *base, IO_REG_TYPE pin)
{
   if (SS_GPIO == GPIO_TYPE(pin)) {
      WRITE_ARC_REG(READ_ARC_REG(base) | (0x01 << GPIO_ID(pin)), base);
   } else {
      MMIO_REG_VAL(base) |= (0x01 << GPIO_ID(pin));
```

```
   }
}

#define DIRECT_READ(base, pin)                  directRead(base, pin)
#define DIRECT_MODE_INPUT(base, pin)            directModeInput(base, pin)
#define DIRECT_MODE_OUTPUT(base, pin)           directModeOutput(base, pin)
#define DIRECT_WRITE_LOW(base, pin) directWriteLow(base, pin)
#define DIRECT_WRITE_HIGH(base, pin)directWriteHigh(base, pin)


#elif defined(ARDUINO_ARCH_STM32)

#define PIN_TO_BASEREG(pin)           (0)
#define PIN_TO_BITMASK(pin)           (pin)
#define IO_REG_TYPE uint32_t
#define IO_REG_ASM

#define DIRECT_READ(base, pin)         digitalRead(pin)
#define DIRECT_MODE_INPUT(base, pin)    pinMode(pin,INPUT)
#define DIRECT_MODE_OUTPUT(base, pin)   pinMode(pin,OUTPUT)
#define DIRECT_WRITE_LOW(base, pin)     digitalWrite(pin, LOW)
#define DIRECT_WRITE_HIGH(base, pin)    digitalWrite(pin, HIGH)

#elif defined(ARDUINO_ARCH_APOLLO3)
#define PIN_TO_BASEREG(pin) (0)
#define PIN_TO_BITMASK(pin) (pin)
#define IO_REG_TYPE uint32_t
#define IO_REG_ASM
#define DIRECT_READ(base, mask) (am_hal_gpio_input_read(mask))
#define DIRECT_MODE_INPUT(base, mask) (am_hal_gpio_pinconfig(mask, g_AM_HAL_GPIO_INPUT))
```

```
#define DIRECT_MODE_OUTPUT(base, mask) (am_hal_gpio_pinconfig(mask, g_AM_HAL_GPIO_OUTPUT))
#define DIRECT_WRITE_LOW(base, mask) (am_hal_gpio_output_clear(mask))
#define DIRECT_WRITE_HIGH(base, mask) (am_hal_gpio_output_set(mask))

#elif defined(ARDUINO_ARCH_RTTHREAD)
#define PIN_TO_BASEREG(pin)            (0)
#define PIN_TO_BITMASK(pin)            (pin)
#define IO_REG_TYPE uint32_t
#define IO_REG_ASM
#define DIRECT_READ(base, pin)         digitalRead(pin)
#define DIRECT_MODE_INPUT(base, pin)    pinMode(pin,INPUT)
#define DIRECT_MODE_OUTPUT(base, pin)   pinMode(pin,OUTPUT)
#define DIRECT_WRITE_LOW(base, pin)     digitalWrite(pin, LOW)
#define DIRECT_WRITE_HIGH(base, pin)    digitalWrite(pin, HIGH)

#endif

// some 3.3V chips with 5V tolerant pins need this workaround
//
#if defined(__MK20DX256__)
#define FIVE_VOLT_TOLERANCE_WORKAROUND
#endif

// library interface description
class CapacitiveSensor
{
  // user-accessible "public" interface
  public:
  // methods
```

```
		CapacitiveSensor(uint8_t sendPin, uint8_t receivePin);
		long capacitiveSensorRaw(uint8_t samples);
		long capacitiveSensor(uint8_t samples);
		void set_CS_Timeout_Millis(unsigned long timeout_millis);
		void reset_CS_AutoCal();
		void set_CS_AutocaL_Millis(unsigned long autoCal_millis);
  // library-accessible "private" interface
  private:
  // variables
		int error;
		unsigned long  leastTotal;
		unsigned int   loopTimingFactor;
		unsigned long  CS_Timeout_Millis;
		unsigned long  CS_AutocaL_Millis;
		unsigned long  lastCal;
		unsigned long  total;
		IO_REG_TYPE sBit;   // send pin's ports and bitmask
		volatile IO_REG_TYPE *sReg;
		IO_REG_TYPE rBit;    // receive pin's ports and bitmask
		volatile IO_REG_TYPE *rReg;
  // methods
		int SenseOneCycle(void);
};

#endif
```
