# Supplementary figures and images for: A Smart Chair to Monitor Sitting Posture by Capacitive Textile Sensors
Source: Materials (Basel). 2023 Jul 5;16(13):4838. doi: 10.3390/ma16134838 (PMC10343373; doi:10.3390/ma16134838)

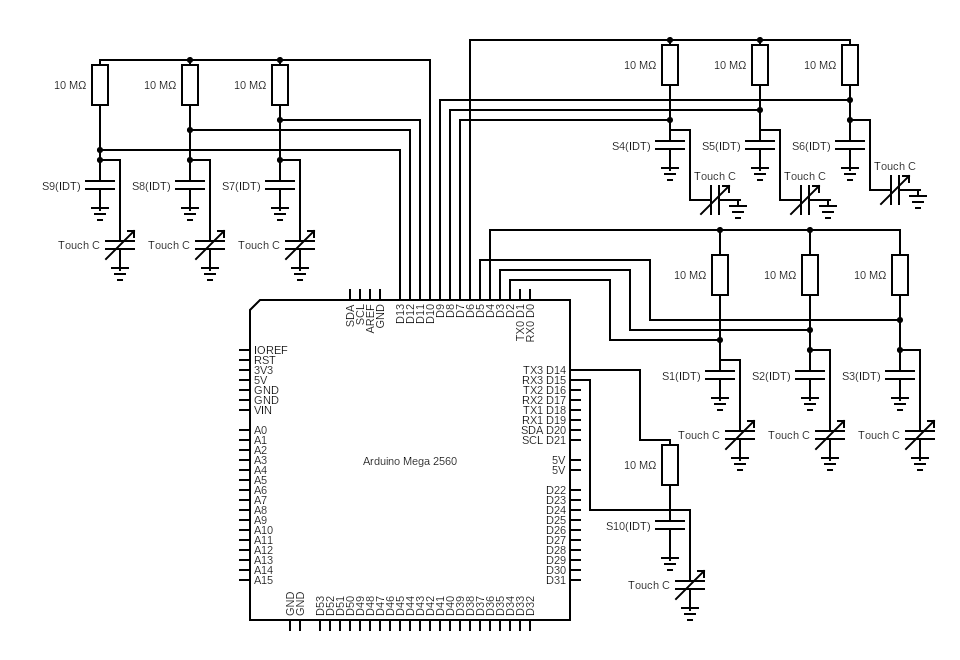

Supplement: Supplementary file 1 [file materials-16-04838-s001.zip › Arduino mega circuit with sensor.png]
